# PCSK9 inhibition interrupts the cross-talk between keratinocytes and macrophages and prevents UVB-induced skin damage

**DOI:** 10.1016/j.jbc.2023.104895

**Published:** 2023-06-06

**Authors:** Chao Luan, Yingxue He, Wen Liu, Yicheng Rong, Jian Gao, Kang Xu, Hui Yu, Yu Hu, Jiaan Zhang, Kun Chen, Wenjie Guo

**Affiliations:** 1 Jiangsu Key Laboratory of Molecular Biology for Skin Diseases and STIs, Institute of Dermatology, Chinese Academy of Medical Sciences & Peking Union Medical College, Nanjing, China; 2 State Key Laboratory of Pharmaceutical Biotechnology, School of Life Sciences, Nanjing University, Nanjing, China

**Keywords:** UVB, PCSK9, STING, keratinocytes, siRNA, SBC110736

## Abstract

Proprotein convertase subtilisin/kexin type 9 (PCSK9) is an enzyme that promotes the degradation of low-density lipoprotein receptors. It is involved in hyperlipidemia as well as other diseases, such as cancer and skin inflammation. However, the detailed mechanism for PCSK9 on ultraviolet B (UVB)-induced skin lesions was not clear. Thus, the role and possible action mechanism of PCSK9 in UVB-induced skin damage in mice were studied here using siRNA and a small molecule inhibitor (SBC110736) against PCSK9. Immunohistochemical staining revealed a significant increase in PCSK9 expression after UVB exposure, indicating the possible role of PCSK9 in UVB damage. Skin damage, increase in epidermal thickness, and keratinocyte hyperproliferation were significantly alleviated after treatment with SBC110736 or siRNA duplexes, compared with that in the UVB model group. Notably, UVB exposure triggered DNA damage in keratinocytes, whereas substantial interferon regulatory factor 3 (IRF3) activation was observed in macrophages. Pharmacologic inhibition of STING or cGAS knockout significantly reduced UVB-induced damage. In the co-culture system, supernatant from UVB-treated keratinocyte induced IRF3 activation in macrophages. This activation was inhibited with SBC110736 and by PCSK9 knockdown. Collectively, our findings reveal that PCSK9 plays a critical role in the crosstalk between damaged keratinocytes and STING activation in macrophages. The interruption of this crosstalk by PCSK9 inhibition may be a potential therapeutic strategy for UVB-induced skin damage.

Solar ultraviolet-B radiation (UVB), with a wavelength range of 280 to 315 nm, is the leading cause of various skin conditions, including photoaging, sunburn, erythema, hyperplasia, inflammation, and melanoma (1, 2, 3). Additionally, the risks of UVB-induced skin damage increase with damage to the ozone layer, creating an urgent need for skin protection against UVB (4). Photoprotective clothing and sunscreens prevent skin redness and sunburn, but they do not relieve or heal sunburned skin (5, 6). However, many antioxidant molecules, such as plant polyphenols, are used to reduce UVB-induced photodamage (7).

The skin is the outermost organ of the human body and is often damaged by environmental factors such as sunlight and air pollution. UVB exposure can lead to increased DNA damage and mutational burden (8). Keratinocytes, in the outermost layer of the skin, act as a shield that absorbs most of the UVB radiation (9). Consequently, UVB-related damage to the skin triggers the keratinocytes to release danger-associated molecular patterns (DAMPs), which can initiate local immune responses, such as antimicrobial peptides (AMPs) and dsDNA (9, 10). Additionally, keratinocytes, which are amateur antigen-presenting cells, express major histocompatibility complex (MHC) class II in several skin disorders characterized by significant T cell infiltration (11).

For the detection of dsDNA, cyclic GMP-AMP (cGAMP) synthase (cGAS) is one of the most critical cytosolic DNA sensors (12). The binding of cGAS to both exogenous and endogenous DNA in the cytoplasm initiates cGAMP synthesis from ATP and GTP. After cGAMP binding, the stimulator of interferon genes (STING) translocates from the endoplasmic reticulum to signaling compartments, where it associates with TANK binding kinase 1 (TBK1). This, in turn, mediates the activation of the transcription factor interferon regulatory factor 3 (IRF3), which initiates the transcription of type I interferons and several interferon-stimulated genes (ISGs) (13). The cGAS–STING signaling pathway has emerged as a key mediator of inflammation in infections, cellular stress, and tissue damage. For example, loss-of-function mutations in TREX1, a DNA repair exonuclease that degrades cytosolic DNA, have been identified in patients with autoimmune disorders such as Aicardi–Goutières syndrome and lupus (14, 15). TREX1 knockout mice exhibit lethal autoimmune destruction of tissues, including the skin (16).

Proprotein convertase subtilisin/kexin type 9 (PCSK9), which functions as a chaperone protein for the LDL receptor, promotes the degradation of LDL receptors and thus increases plasma LDL concentration (17, 18). Clinical trials have shown that the inhibition of PCSK9 by siRNA or neutralizing antibodies maintains or elevates the expression of LDL receptors and contributes significantly to lowering blood cholesterol levels (19). In addition to regulating the expression of LDL receptors, PCSK9 also regulates other proteins on the cell surface. A recent study showed that PCSK9 inhibition increased the expression of MHC protein class I proteins on the tumor cell surface. Moreover, another study reported the role of PCSK9 in regulating macrophage apoptosis and pro-inflammatory cytokine secretion (20).

We previously reported that PCSK9 is overexpressed in psoriatic lesions and that its inhibition can decrease the inflammatory response and hyperproliferation of keratinocytes induced by imiquimod treatment (21). Furthermore, a recent study revealed an association between circulating PCSK9 and early and advanced stages of atherosclerosis in psoriasis (22). However, we lack reports on the function of PCSK-9 in UVB-induced skin damage. Thus, in this study, we examined the role of PCSK9 in UVB-induced skin damage as well as the topical and pharmacological inhibition of PCSK9 using siRNA and SBC110736, respectively, as a potential treatment for this damage.

## Results

### UVB light induces PCSK9 expression in mouse skin

**Figure 1:**
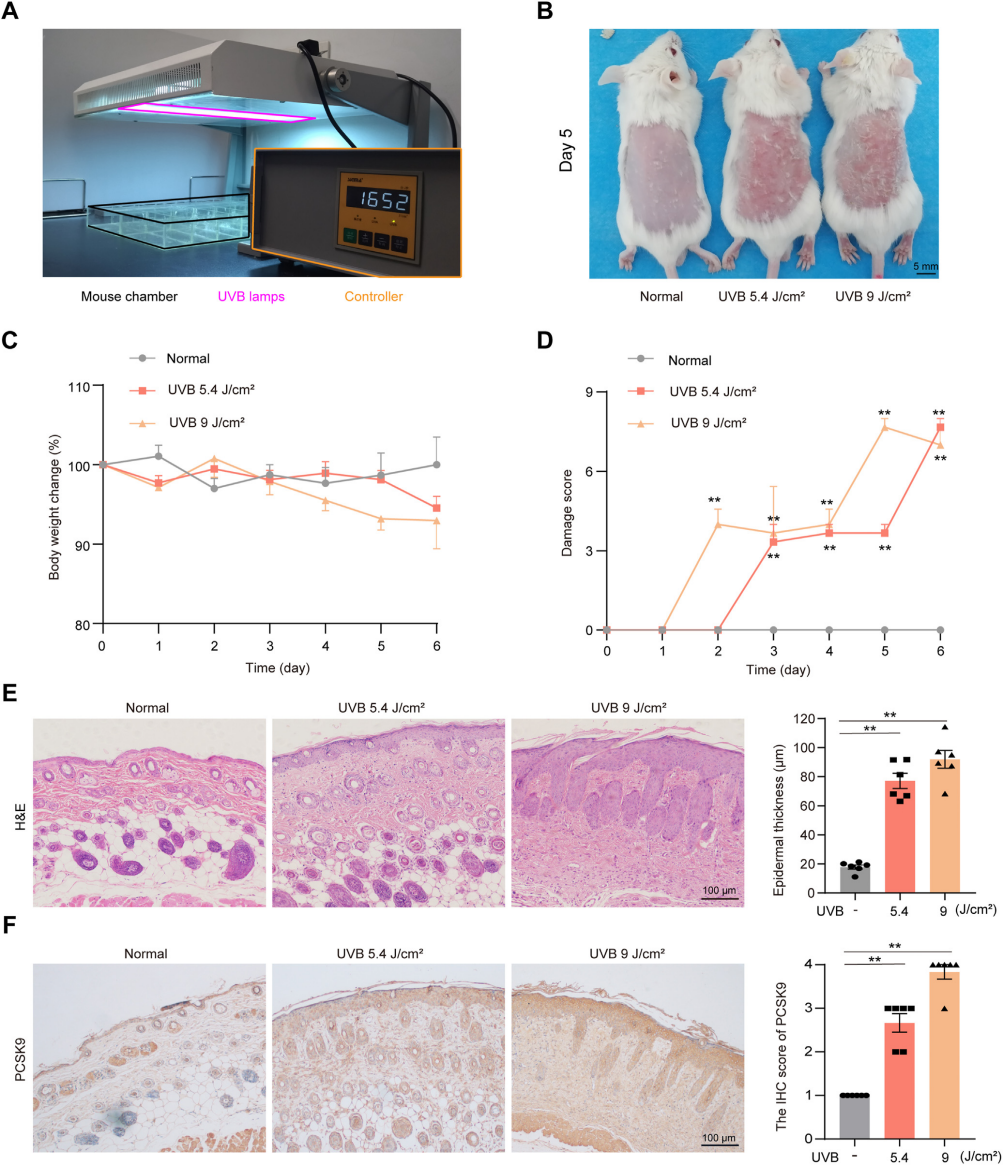
**UVB triggered significant damage to the skin in mice accompanied by elevated PCSK9 expression.** The hair on the backs of the female BALB/C mice (6–8 weeks old) was removed 2 to 3 days before the UVB-irradiation experiments. The shaved skin was daily applied with a dose of 5.4 J/cm^2^ or 9 J/cm^2^ (UVM-225D Mineralight) once a day for 5 days. *A*, Scheme for UVB exposure. *B*, Representative photos of dorsal skin after 5 days of UVB exposure. *C*, The body weight changes in mice during the experiment. *D*, Damage scores of skin during UVB treatment. *E*, H&E staining of dorsal skin in mice. *F*, IHC staining of PCSK9. Scale bar: 100 μm. Data in b and c were expressed as means ± SD of six mice. Static data in E and F were expressed as means ± SD of five fields per mouse in each group. n = 6 mice per group. ∗*p* < 0.05, ∗*p* < 0.01 *versus* normal group.

Initially, we established a UVB light-induced skin damage model and examined the expression of PCSK9 where mice were exposed to two doses of UVB irradiation (Fig. 1*A*). Compared with the unexposed (normal) group, UVB exposure caused a gradual and dose-dependent increase in skin damage in mice, ranging from erythema and bleeding to dryness and scarring, all the way to irritation and skin erosion. No significant difference in body weight was observed between these groups (Fig. 1, *B*–*D*). After 5 days of UVB irradiation, H&E staining revealed significant thickening of the epidermis (Fig. 1*E*). Additionally, immunohistochemical staining revealed that exposure to UVB elevated PCSK9 expression (Fig. 1*F*). Thus, UVB exposure caused significant skin damage, and the lower but effective irradiation dose of 5.4 J/cm^2^ was chosen for subsequent experiments.

### Topical application of siRNA inhibits UVB-induced skin damage *via* PCSK9 and proliferating cell nuclear antigen suppression

**Figure 2:**
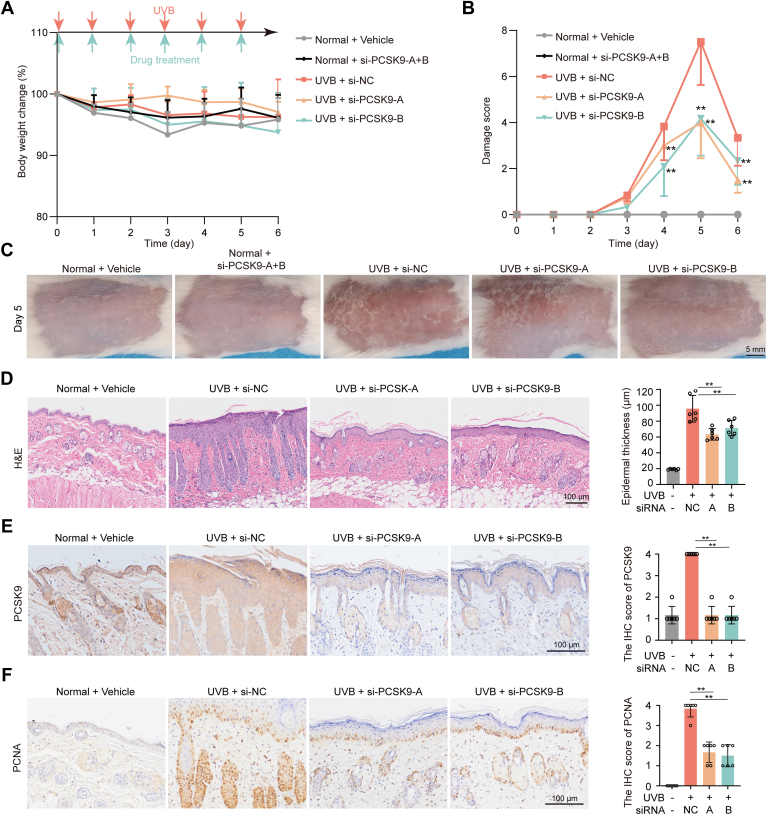
**Topical application of PCSK9 siRNA reduced UVB-induced skin damage *in vivo*.** The hair on the backs of the female BALB/C mice (6–8 weeks old) was removed 2 to 3 days before the UVB-irradiation experiments. The shaved skin was daily applied with a dose of 5.4 J/cm^2^ (UVM-225D Mineralight) for 5 days. Si-NC or si-PCSK9-A or si-PCSK9-B in gel emulsifier were given to mice once a day for 5 days. *A*, The scheme and the bodyweight change of mice during UVB treatment. *B*, The damage score of mice during the experiment. *C*, Representative photos of dorsal skin. *D*, H&E staining of dorsal skin. *E*, IHC staining of PCSK9. *F*, IHC staining of PCNA. All the data were expressed as means ± SD, n = 6. The data in d are shown as the means ± SD of five fields of view per mouse in every group, n = 6 mice per group. ∗*p* < 0.05, ∗∗*p* < 0.01 *versus* UVB + si-NC or as indicated. Scale bar: 100 μm.

To confirm the correlation between PCSK9 and UVB-induced skin damage, siRNA-mediated PCSK9 inhibition was tested. Two siRNA duplexes targeting mouse PCSK9 (si-RNA-A and si-RNA-B) and a nonsense mouse control siRNA (si-RNA-NC) were designed and used. Normal mice that received si-RNA-A and si-RNA-B showed no changes in skin damage score and body weight, indicating good tolerance to this treatment (Fig. 2, *A*–*C*). Simultaneously, mice exposed to UVB exhibited a significant increase in skin damage score by day 2 and peaked on day 5. In contrast, the two treatment groups, UVB with si-RNA-A and UVB with si-RNA-B, showed a significant reduction in damage scores by day 4 of UVB exposure, which lasted until day six (Fig. 2, *B* and *C*). Concordantly, the expression of PCSK9 in the epidermis was significantly reduced in the treatment groups (Fig. 2*E*).

Treatment with siRNA significantly reduced the thickness of the epidermis (Fig. 2*D*). Next, immunohistochemistry (IHC) was used to investigate the expression of proliferating cell nuclear antigen (PCNA), which is involved in skin cell proliferation. After treatment with siRNA, PCNA expression was significantly reduced (Fig. 2*F*). This indicates that PCSK9 knockdown reduces skin epidermal cell proliferation caused by UVB radiation.

### Pharmacologic inhibition of PCSK9 reduces UVB-induced skin damage

**Figure 3:**
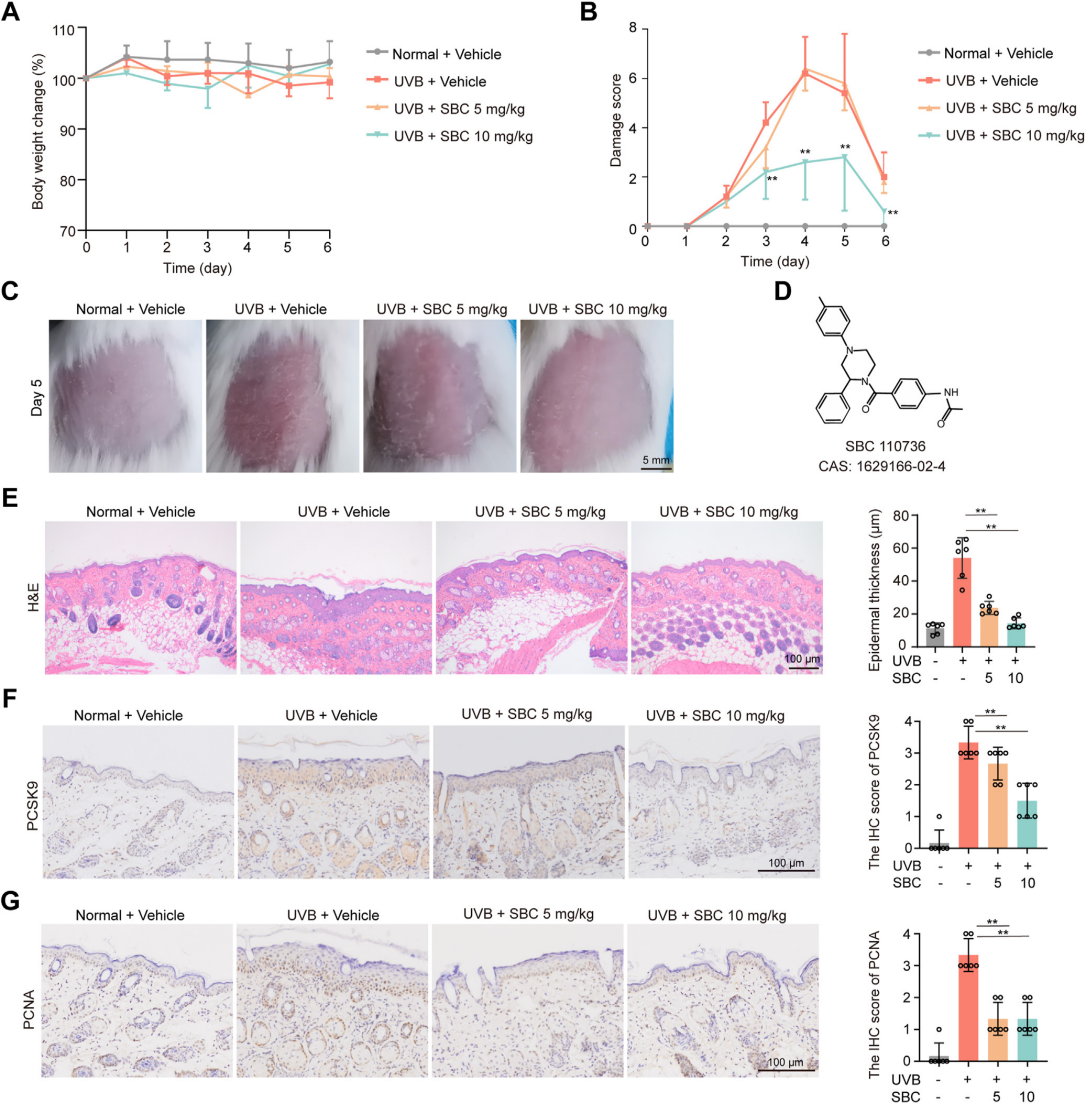
**Pharmacologic inhibition of PCSK9 reduced UV-induced skin damage.** The hair on the backs of the BALB/C mice (6–8 weeks old) was removed 2 to 3 days before the UVB-irradiation experiments. The shaved skin was daily applied with a dose of 5.4 mJ/cm^2^ (UVM-225D Mineralight) for 5 days. PCSK inhibitor (PCSKi) SBC110736 was given to mice (i.p.) once a day for 5 days. *A*, The body weight changes of mice during the experiment. *B*, The damage score of skin during the experiment. *C*, Representative photos of dorsal skin. *D*, Chemical structure of PCSK9 inhibitor SBC110736 (PCSK9i). *E*, Representative H&E image of dorsal skin and thickness of the epidermis on day 6. *F*, Representative IHC staining image of PCSK9 expression on day 6. *G*, IHC staining of PCNA. Static data in (*E*), (*F*), and (*G*) were expressed as means ± SD of five fields per mouse in each group. n = 6 mice per group. ∗*p* < 0.05, ∗*p* < 0.01 *versus* as indicated. Scale bar: 100 μm.

To further explore the role of PCSK9 in skin damage and epidermal hyperproliferation caused by UVB radiation, SBC110736, an inhibitor of PCSK9, was employed (23) (Fig. 3*D*). No significant difference in weight change was observed after SBC110736 treatment (Fig. 3*A*). Compared with the model group, the skin damage score (Fig. 3, *B* and *C*) and epidermis thickness (Fig. 3*E*) were significantly decreased after SBC110736 treatment. Next, IHC analysis of tissue sections revealed a significant increase in PCSK9 and PCNA expression after UVB irradiation compared with the normal group. This expression decreased after SBC treatment (Fig. 3, *F* and *G*). Taken together, siRNA-mediated PCSK9 knockdown and pharmacological inhibition of PCSK9 reduce UVB-induced skin damage.

### PCSK9 knockdown and SBC110736 treatment inhibit UVB-induced activation of the cGAS-STING pathway

**Figure 4:**
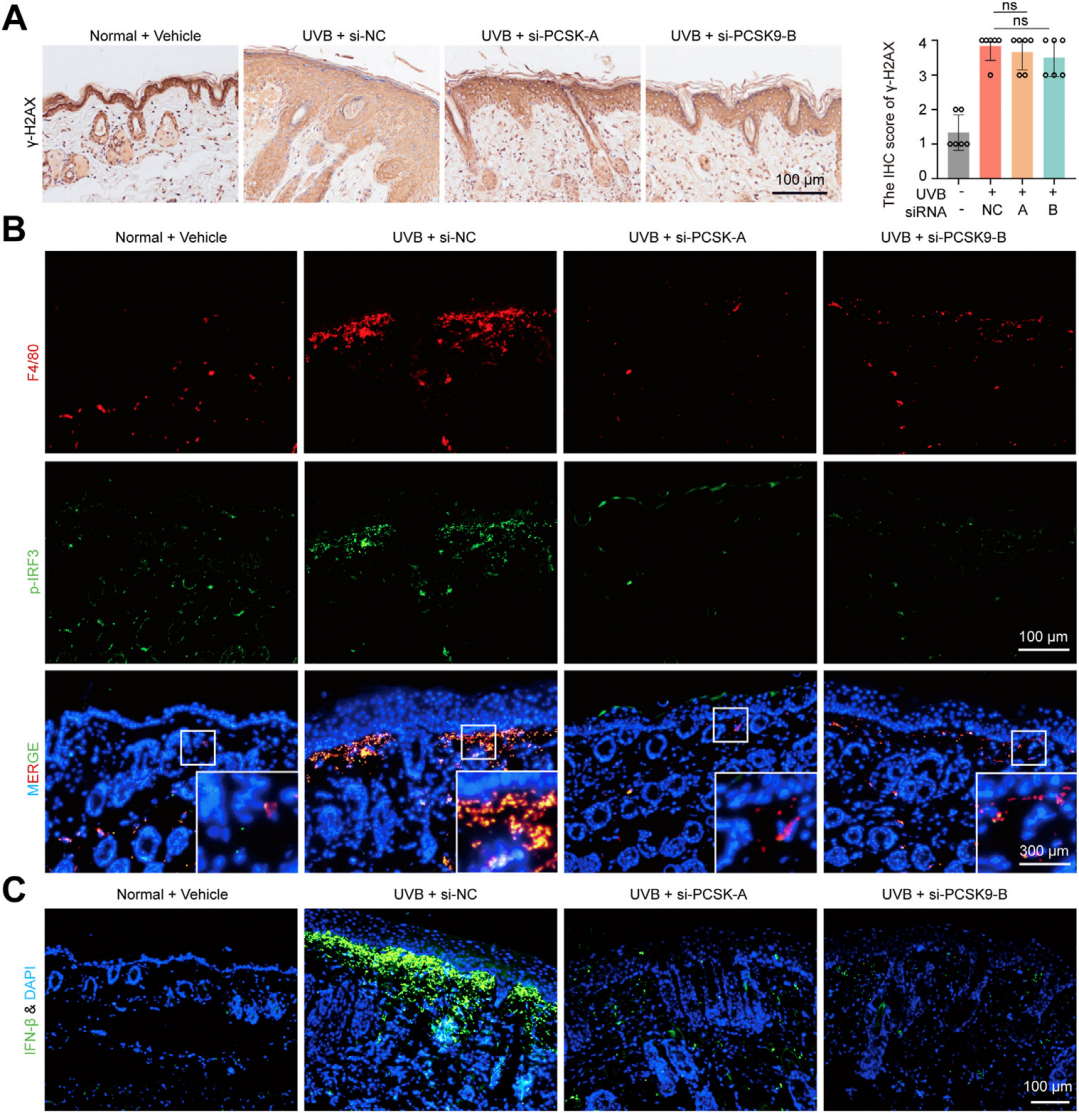
**Topical application of PCSK9 siRNA reduced UVB-induced IRF3 activation in macrophage.***A*, Representative image of γH2AX expression at day 6. *B*, Co-localization of macrophages marked by F4/80 and p-IRF3. *C*, Representative image of IFN-β expression. Data were shown as the means ± SD of five fields of view per mouse in every group, n = 6 mice per group. ∗*p* < 0.05, ∗∗*p* < 0.01 *versus* as indicated. Scale bar: 100 μm.

**Figure 5:**
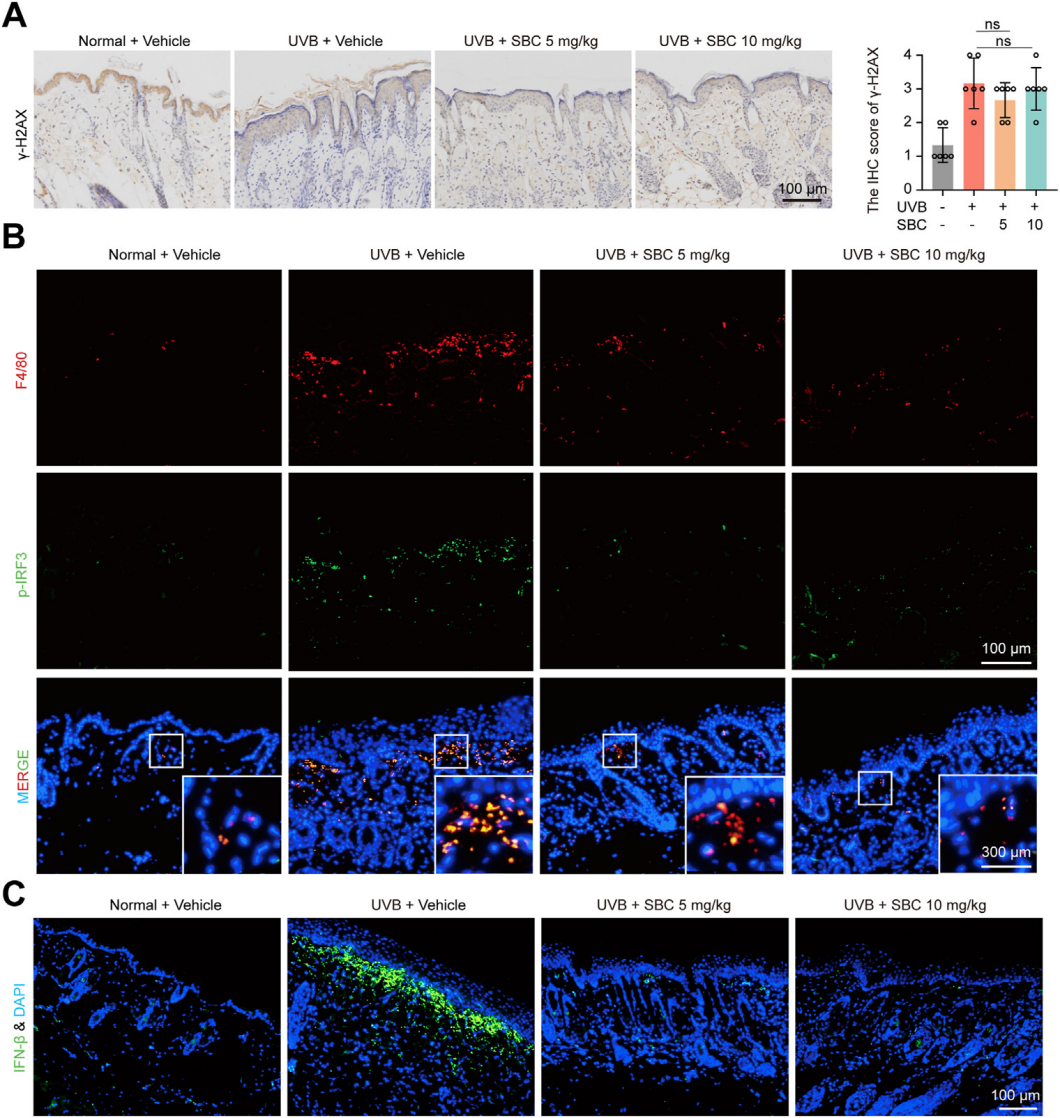
**Pharmacologic inhibition of PCSK9 reduced UVB-induced IRF3 activation in macrophages.***A*, Representative image of γH2AX expression. *B*, Co-localization of macrophages marked by F4/80 and p-IRF3. *C*, Representative image of IFN-β expression. Data were shown as the means ± SD of five fields of view per mouse in each group, n = 6 mice per group. ∗*p* < 0.05, ∗∗*p* < 0.01 *versus* as indicated. Scale bar: 100 μm.

UVB irradiation leads to DNA damage and the accumulation of cytosolic dsDNA, the initial activator of the STING pathway. Phosphorylation of histone H2AX (γH2AX) on serine 139 is associated with DNA double-strand breaks, a hallmark of DNA damage (24). IHC showed that UVB radiation increased γH2AX-positive cells, whereas si-RNA and SBC110736 treatment showed no significant inhibitory effect (Figs. 4*A* and 5*A*). Next, we used immunofluorescence (IF) to investigate the expression of phosphorylated IRF3 (p-IRF3), a transcription factor in the cGAS-STING pathway. Our data revealed a significant positive correlation between UVB irradiation and cGAS-STING pathway activation marked by the expression of p-IRF3 (Figs. 4*B* and 5*B*) and IFN-β (Figs. 4*C* and 5*C*). These, in turn, were significantly inhibited by si-PCSK9 and SBC110736. IHC analysis confirmed these results further (Fig. S1). Notably, the expression of p-IRF3 and p-TBK1 was concentrated in the dermis and expressed in macrophages, which were marked by F4/80 (Figs. 4*B* and 5*B*) and CD11b (Fig. S2) (25). These results indicate that the cGAS-STING pathway is mainly activated in immune cells, rather than in keratinocytes.

### The STING inhibitor C176 and cGAS knockout mice inhibit UVB-induced skin damage

**Figure 6:**
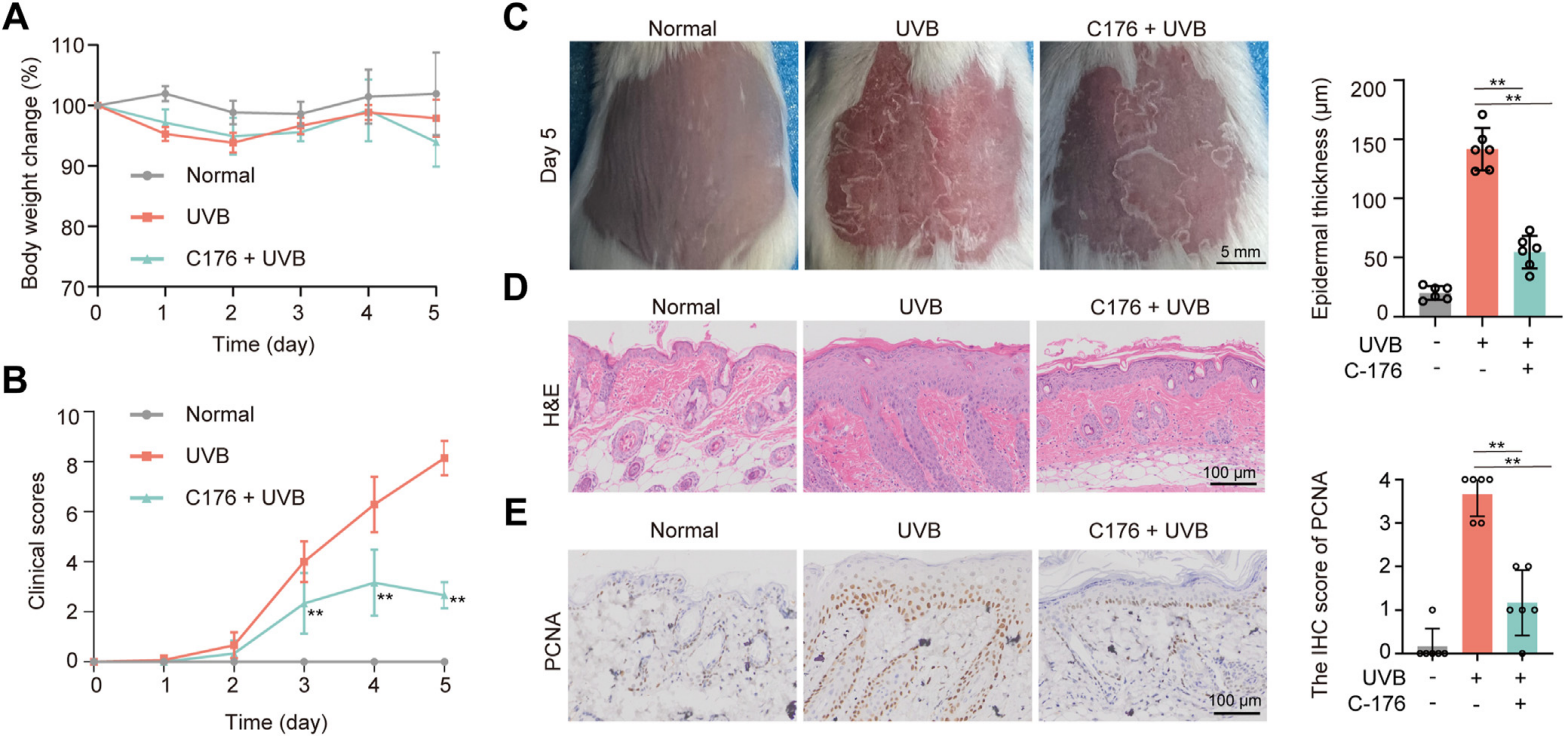
**Pharmacologic inhibition of STING reduced UVB-induced damage.***A*, The body weight changes of mice during UVB treatment. *B*, The damage score of mice during the experiment. *C*, Representative photos of dorsal skin in mice. *D*, Representative H&E image of dorsal skin and thickness of the epidermis in mice at day 5. *E*, Representative IHC image of PCNA expression on day 5. Data were shown as the means ± SD of five fields of view per mouse in every group, n = 6 mice per group. ∗*p* < 0.05, ∗∗*p* < 0.01 *versus* as indicated.

**Figure 7:**
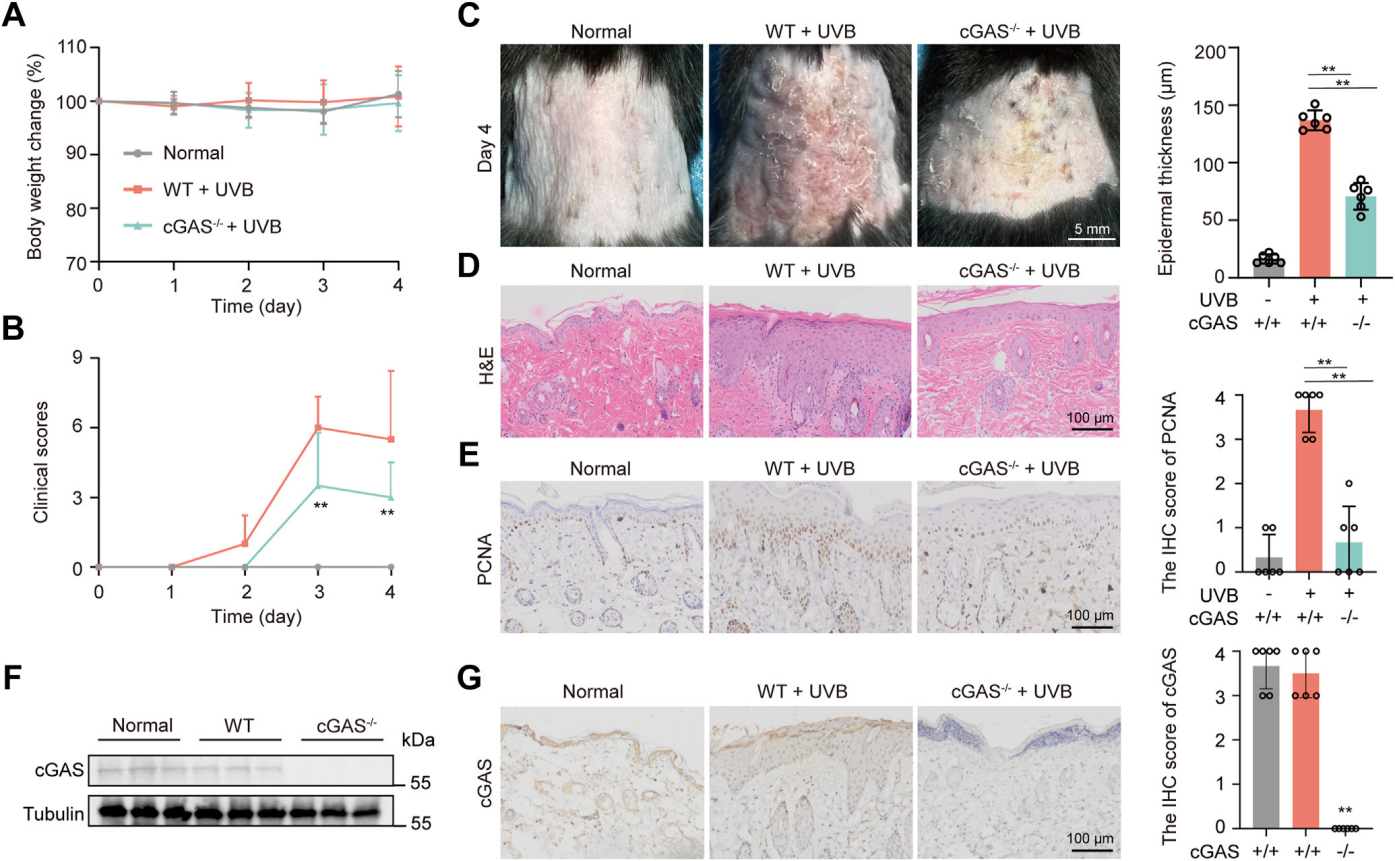
**Deficiency in cGAS reduced UVB-induced damage.***A*, The body weight changes of mice during UVB treatment. *B*, The damage score of mice during the experiment. *C*, Representative photos of dorsal skin in mice. *D*, Representative H&E image of dorsal skin and thickness of the epidermis in mice at day 4. *E*, Representative image of PCNA expression on day 4. *F*, Expression of cGAS protein in cGAS knockout mice. *G*, Representative IHC image of cGAS expression. Data were shown as the means ± SD of five fields of view per mouse in every group, n = 6 mice per group. ∗*p* < 0.05, ∗∗*p* < 0.01 *versus* as indicated.

To confirm the role of the cGAS-STING pathway in UVB-induced skin damage, the effects of the small molecule STING inhibitor C176 and cGAS knockout mice were examined. As in Figure 6, skin damage score and epidermis thickness improved significantly after C176 treatment compared with that in the model group. Likewise, the C176 treatment reduced UVB-induced PCNA expression (Fig. 6*E*). Similarly, cGAS knockout mice exhibited resistance to UVB-induced damage as evidenced by decreased skin damage score, epidermis thickness, and PCNA expression compared with that in wild-type mice (Fig. 7). This confirms that cGAS-STING pathway activation contributes to UVB-induced skin damage.

### PCSK9 did not affect DNA damage, damage repair, and STING activation in HaCaT cells

Based on the earlier results, we proposed that UVB irradiation causes DNA damage in keratinocytes in the skin and activates the cGAS-STING pathway, to a greater extent, in nearby immune cells. To confirm this hypothesis, a UVB-irradiated cell model (26) was established using HaCaT cells. HaCaT cells were irradiated with 50 mJ/cm^2^ UVB light and harvested various times after UVB irradiation. The expression of γH2AX protein increased in a time-dependent manner after UVB treatment from 30 min to 10 h in HaCaT cells. However, the expression of p-IRF3 and p-TBK1 remained unaltered, suggesting that the cGAS-STING pathway in keratinocytes was not activated, although DNA damage occurred (Fig. S4*A*). We chose the colon cancer cell line HCT-116 as a positive control for cGAS-STING pathway activation (13). Thus, in contrast to HaCaT cells, DNA damage and the cGAS-STING pathway activation were observed in HCT-116 cells following UVB irradiation (Fig. S4*B*).

Next, we examined the effects of PCSK9 on DNA damage and repair. HaCaT cells were pretreated with SBC110736 2 h before UVB irradiation at 50 J/m^2^ and harvested after different hours of UVB irradiation. The expression level of the γ-H2AX protein was not significantly different between the two groups, suggesting that PCSK9 did not alter UVB-induced DNA damage (Fig. S4, *C* and *D*). The effect of PCSK9 on two different DNA repair pathways was examined using two well-characterized GFP-based reporting systems (Fig. S5*A*). No significant changes in HR and NHEJ pathways were observed after SBC110736 treatment (Fig. S5, *B* and *C*).

### PCSK9 regulates dsDNA release from keratinocytes and activates the STING pathway in macrophages

**Figure 8:**
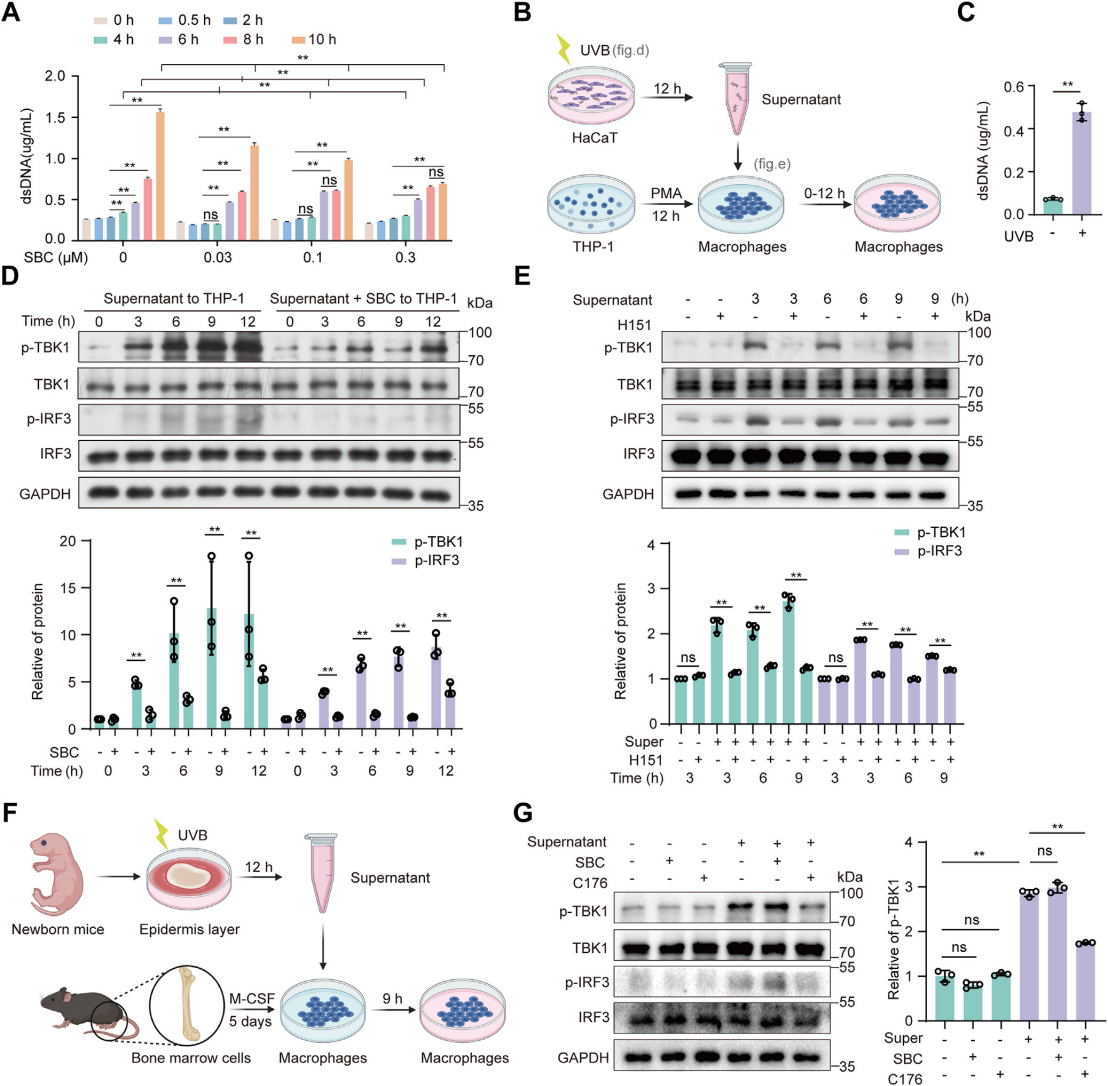
**PCSK9 inhibition reduced UVB-induced dsDNA release from keratinocytes and decreased STING pathway activation in macrophages.***A*, HaCaT cells pretreated with SBC110736 for 2 h were irradiated with UVB (50 mJ/cm^2^), and supernatants were collected at corresponding time points. The concentration of extracellular dsDNA in the culture supernatant was detected using the PicoGreen dsDNA kit. *B*, Operation flow chart. HaCaT cells were cultured for 12 h after UVB irradiation and the supernatant was used for further incubation of PMA-differentiated THP-1 cells. *C*, The concentration of extracellular dsDNA in the HaCaT culture supernatant at 12 h after UVB irradiation. *D*, Supernatant from SBC110736 (0.3 μM)-treated HaCaT cells after irradiation loses the ability to activate STING in PMA-differentiated THP-1 cells. *E*, The STING inhibitor H151 inhibited supernatant-induced STING activation in macrophages (PMA-differentiated THP-1 cells). *F* and *G*, Supernatant-activated STING in macrophages (BMDMs) was inhibited by H151 but not SBC110736. The skin tissue of the newborn mice was collected and digested overnight with the dispase Ⅱ enzyme at 4 °C to obtain the epidermis layer which was then irradiated with 50 mJ/cm^2^ UVB. After 12 h, the supernatant containing dsDNA was collected and used to stimulate BMDMs. Data were shown as the means ± SD in every group. ∗*p* < 0.05, ∗∗*p* < 0.01 *versus* as indicated. Ns, not significant.

To further confirm how PCSK9 affects STING pathway activation in immune cells, the effects of SBC110736 on the release of dsDNA from UVB-treated keratinocytes cells and dsDNA-induced STING activation in macrophages were examined. The culture supernatant from UVB-irradiated HaCaT cells was collected, and the concentration of extracellular dsDNA was detected using the PicoGreen dsDNA kit. As shown in Figure 8*A*, dsDNA accumulated in the culture supernatant of the UVB-treated HaCaT cells. The treatment of HaCaT cells with SBC110736 significantly reduced dsDNA release into the culture supernatant (Fig. 8*A*). The supernatant of HaCaT collected 12 h after UVB irradiation was used for further incubation with THP-1-derived macrophages (Fig. 8*B*). The concentration of extracellular dsDNA in the culture supernatant at 12 h after UVB irradiation (50 mJ/cm^2^) was 0.47 μg/ml (Fig. 8*C*). The culture supernatant from UVB-irradiated HaCaT cells activated the STING pathway in THP1-derived macrophages, which was inhibited when HaCaT cells were treated with SBC110736 (Fig. 8*D*). Additionally, the human STING inhibitor H151 was found to inhibit this STING pathway activation (Fig. 8*E*). DNase (2 U/ml) was used to degrade dsDNA in the supernatant; this reduced the activation of STING in THP-1 cells (Fig. S6*A*). In contrast, SBC110736 treatment of THP-1 cells did not affect HaCaT supernatant-induced STING activation in THP-1 cells (Fig. S6*B*).

At the same time, we obtained similar conclusions in mouse cells. UVB irradiation of mouse epidermal cells results in the secretion of dsDNA, which can activate the STING signaling pathway in bone marrow-derived macrophages (BMDMs) and is inhibited by a mouse-specific STING inhibitor (C176) but not by SBC110736 (Fig. 8, *F* and *G*).

These results indicate that inhibition of PCSK9 reduced the release of dsDNA from UVB-induced keratinocytes. Moreover, the dsDNA released by the keratinocytes can activate the STING signaling pathway in macrophages.

## Discussion

When exposed to solar radiation, high-energy UVB can penetrate the epidermis, where it is mainly absorbed by keratinocytes (27). UVB radiation produces large numbers of cyclobutene pyrimidine dimers, which stress cells by interfering with DNA replication and transcription (28). In addition, it can induce various types of oxidative DNA damage and single-stranded DNA breaks, producing DNA fragments released in extracellular vesicles that induce STING-mediated inflammatory response in other cells (29, 30). Our results demonstrate that dsDNA-containing supernatants generated by HaCaT cells after UVB irradiation can activate STING in THP-1 cells. At the same time, STING activation in THP-1 cells was attenuated by treatment with DNase. Recent evidence suggests that STING activation can transfer between cells and promote anti-tumor immunity (30). However, our results demonstrated that UVB-induced activation of STING in HaCaT was weak. In this study, we observed that activation of the STING pathway contributed to UVB-induced photodamage. This was evidenced by the improvement in the skin condition with the use of the STING pharmacological inhibitor C176 or knocking out the *cGAS* gene. As STING plays a crucial role in the antiviral immune response (31), direct inhibition of STING to reduce sunburns may not be a good approach.

Several studies have shown that PCSK9 plays a vital role in inflammatory disease (20). However, the correlation between PCSK9 and skin lesions was rarely reported. Our results indicated that inhibiting PCSK9 expression using RNA interference or pharmacological inhibitor can prevent UVB-induced skin damage. Moreover, this protective effect was not achieved by reducing DNA damage or increasing the efficiency of DNA double-strand repair in keratinocytes (Figs. S4 and S5). Conversely, PCSK9 inhibition likely hindered the transport of dsDNA from keratinocytes to immune cells (Fig. 8). A distinct finding that emerged from our analysis was that DNA damage occurred in the epidermis. In contrast, broken DNA activated the STING pathway in immune cells rather than in keratinocytes. A possible explanation might be that vitamin D is produced in keratinocytes after UVB irradiation, inhibiting STING activation (32). Since UVB can only penetrate the epidermis, it cannot cause DNA damage in immune cells, which are localized in the dermis, especially if the epidermis is thickened (33). Therefore, STING pathway activation in immune cells is caused by broken DNA fragments released by keratinocytes. This activation of STING causes immune cells to release various pro-inflammatory cytokines, which promote keratinocyte proliferation. We observed that UVB-induced dsDNA release in keratinocytes was reduced by a pharmacological inhibitor of PCSK9, which can explain the consequent reduction in the thickening of the epidermis as well (Fig. 3).

Besides the cGAS-STING pathway, there are other DNA-sensing pathways in the body, such as the AIM2 inflammasome and TLR9 (34). As shown, the expression of IFN-β and p-IRF3 in macrophages was almost completely abrogated (Fig. S3, *A*–D). These findings suggest STING inhibitors and cGAS gene knockout can effectively inhibit the activation of the cGAS-STING pathway. However, despite this inhibition, the skin injury phenotype in mice was not completely alleviated, as observed in Figures 6 and 7. This incomplete alleviation is likely since the recognition of dsDNA is mediated by multiple signaling pathways *in vivo*. After applying STING inhibitors and cGAS gene knockout, the expression of GSDMD and IL-1β is still detected in the skin tissue, as depicted in Fig. S3, *E* and *F*. This may imply a combination strategy for inhibiting DNA-sensing pathways for eliminating UVB-induced skin damage.

In conclusion, this study helps shed light on some of the pathological mechanisms of sunburn. Additionally, it provides a potential therapeutic approach for the prevention and treatment of sunburn without damaging the anti-viral ability of the body. These observations have several implications for future research on the role of DAMPs in dermatological diseases, such as psoriasis and systemic lupus erythematosus. Moreover, how PCSK9 affects dsDNA release is still to be studied next.

## Experimental procedures

### Reagents

Anti-PCSK9 (SC-55206), anti-GSDMD (L0815), and anti-PCNA (sc-56) were purchased from Santa Cruz Biotechnology. Anti-γH2AX (80312), anti-p-IRF3 (37829S), anti-IL-1β (12242S), and anti-p-TBK1 (38066) antibodies were purchased from Cell Signaling Technology. The anti-GAPDH (M20005) antibody was purchased from Abmart. The anti-cGAS (A8335) antibody and 488-conjugated Goat Anti-Mouse IgG (H + L) (AS037) were purchased from ABclonal. Goat anti-Mouse IgG (H + L) Cross-Adsorbed Secondary Antibody, Alexa Fluor 546 (A11003) was purchased from Invitrogen The Hieff Trans Liposomal Transfection Reagent (40802ES02) and Dispase Ⅱ (40104E) were purchased from YEASEN (Shanghai, China). Anti-IFN-β (YT5964) was purchased from Immunoway. CD11b-PE (B253922) and Anti-F4/80-PE (123110) were purchased from Biolegend. SBC-110736 (T4524), H-151 (T5674), and C176 (T5154) were obtained from TargetMol. The Immunohistochemical (IHC) analysis kit (3,3ʹ-diaminobenzidine) was purchased from Proteintech.

### Mice

BLAB/C (female, 6–8 weeks, 18–22 g), C57BL/6J (female, 6–8 weeks, 18–22 g), and C57BL/6J (born within 24 h) were purchased from Jiangsu Gempharmatech Co, Ltd cGAS^−/−^ mice were obtained from Prof. Cheng Qian of Nanjing Medical University. Mice were maintained in an animal facility under standard laboratory conditions for 1 week before the experiments and provided water and standard chow. The animal welfare and experimental procedures were approved by the Institutional Animal Ethnical and Welfare Committee of Nanjing University (IACUC-D2003011, Nanjing, China). All efforts were made to reduce the number of animals used and to minimize animals’ suffering.

### Cells and culture

Human immortalized keratinocyte cell lines (HaCaT) were purchased from the Ethics Committee of the Kunming Institute of Zoology, Chinese Academy of Sciences. Human colon cancer cell line (HCT-116), human embryonic kidney cell line (HEK-293), and human acute monocytic leukemia cell line (THP-1) were purchased from Shanghai Cell Bank of Chinese Academy. These cells were cultured in DMEM high-glucose (Biological Industries) or RPMI1640 medium (Gibco) supplemented with 10% fetal bovine serum (Biological Industries), 100 μg/ml of streptomycin, and 100 U/ml of penicillin, and cultured with 5% CO_2_ at 37 °C in a humidified atmosphere.

### UVB exposure

HaCaT cells were irradiated with UVB light at a dosage of 50 mJ/cm^2^ by using UVB lamps (SS07-T, Sigma, China). To avoid UVB absorption by the medium, cell layers were washed with PBS twice and covered with 200 μl of PBS per well in 6-well plates when exposed to UVB irradiation.

### UVB-induced skin damage

The UVB-induced skin damage model in mice was done as reported (35). The hair on the backs of the mice was removed 2 to 3 days before the UVB-irradiation experiments. The shaved skin was exposed to UVB light (30 mW/cm^2^) for 5.4 J/cm^2^ (3 min) or 9 J/cm^2^ (5 min) every day for 5 consecutive days. The clinical severity of mouse skin lesion (dermatitis) was scored using the macroscopic diagnostic criteria normally used when assessing the severity of dermatitis in humans. The severity of dermatitis was evaluated every day as reported (36). For skin conditions such as erythema/bleeding, scar/dryness, and scratching/erosion, according to their respective clinical severity of skin scores: 0 (none), 1 (mild), 2 (moderate), and 3 (severe), all added together, the total score is 9. The sum of the individual score was taken as the dermatitis score.

### Animal treatment

For siRNA topical treatment, siRNA duplexes targeting mouse Pcsk9 (si-RNA-A and si-RNA-B) and nonsense mouse control siRNA (si-NC) were designed and applied as reported (21). Duplexes were suspended in a 3:5 mixture of cream emulsifier (Johnson’s baby lotion; Johnson & Johnson) and water at a final concentration of 12.5 μM. The shaved region was treated with 30 μl si-PCSK9-A (n = 6), si-PCSK9-B (n = 6) or siRNA-NC (n = 6) once per day for five consecutive days. One hour after the siRNA treatment, UVB treatment was applied.

For PCSK9 pharmacologic inhibitor (SBC 110736) treatment, wild-type mice were injected intraperitoneally with 10 mg/kg SBC110736 once per day for 5 consecutive days. SBC110736 was dissolved in the vehicle (10% DMSO, 30% PEG 400, 5% Tween 80 in PBS).

For the STING pharmacologic inhibitor studies, wild-type mice were injected intraperitoneally with 750 nmol C-176 per mouse in 200 μl corn oil (TargetMol) (37) once per day for five consecutive days.

### Histology, IHC, and IF assay

Skin samples were embedded in paraffin. Sections (4 μm thick) of each specimen were cut for hematoxylin and eosin staining, IHC, and IF study. For H&E stain, the epidermis thickness of H&E-stained skin samples from 20 different locations was measured, and the average thickness for each mouse was calculated. IHC and IF staining was performed as previously reported (38).

Images were evaluated *via* IHC Optical density score. First, we used image J (NIH) and its plug-in IHC Profiler to quantitatively analyze the IHC result image to automatically score the image to obtain semi-quantitative results (high positive, positive, low positive, or negative). Then, using the method reported in the literature (39), we calculated the optical density score (from 0 to 4) of the IHC image using the resulting algebraic formula of Image J.

### Quantification of dsDNA

Levels of dsDNA were detected by using the PicoGreen assay Kit (Life Technologies) as previously described (38). Briefly, dsDNA in cell culture medium was collected and mixed 1:1 with the PicoGreen, and fluorescence was then measured (ex: 485 nm, em: 528 nm). The DNA concentrations were calculated using a standard curve.

### The cell supernatant containing dsDNA extraction from HaCaT and mouse keratinocytes and *in vitro* stimulation

HaCaT cells were cultured for 12 h after 50 mJ/cm^2^ UVB irradiation. The cell supernatant containing dsDNA was used to culture PMA-differentiated THP-1 cells.

The skin tissue from newborn mice was digested overnight with dispase II enzyme at 4 °C to isolate the epidermis layer, which is mainly composed of keratinocytes (40). After irradiating the epidermis layer of mice with 50 mJ/cm^2^ UVB radiation, 1 ml of complete DMEM medium was added, and the mixture was cultured in an incubator for 12 h. The cell supernatant containing dsDNA was used to culture BMDMs that were differentiated by MCSF as reported (41).

### DNA damage repair assays

DNA damage repair including HR and non-homologous end joining (NHEJ) repair in HACAT cells was detected as reported (13). Briefly, 5 × 10^5^ HaCaT cells were co-transfected with 2 μg pCBASce or empty pcDNA vector, and either 4 μg pimEJ5-GFP or pHPRT-DR-GFP plasmid. GFP expression in cells was analyzed using FACS at 48 h.

### Statistical analysis

All data were analyzed using GraphPad Prism Software Version 8.0 (GraphPad Software) and expressed as means ± SD. Student tests and one-way ANOVA were used for data analysis. *p* < 0.05 is considered a significant difference.

## Data availability

All data are contained within the manuscript.

## Supporting information

This article contains supporting information.

## Conflict of interest

The authors have no conflicts of interest to declare.
