# Supplementary material for: PCSK9 inhibition interrupts the cross-talk between keratinocytes and macrophages and prevents UVB-induced skin damage
Source: J Biol Chem. 2023 Jun 6;299(7):104895. doi: 10.1016/j.jbc.2023.104895 (PMC10331474; doi:10.1016/j.jbc.2023.104895)
Supplement: Supporting Figures S1–S6 [file mmc1.pdf]

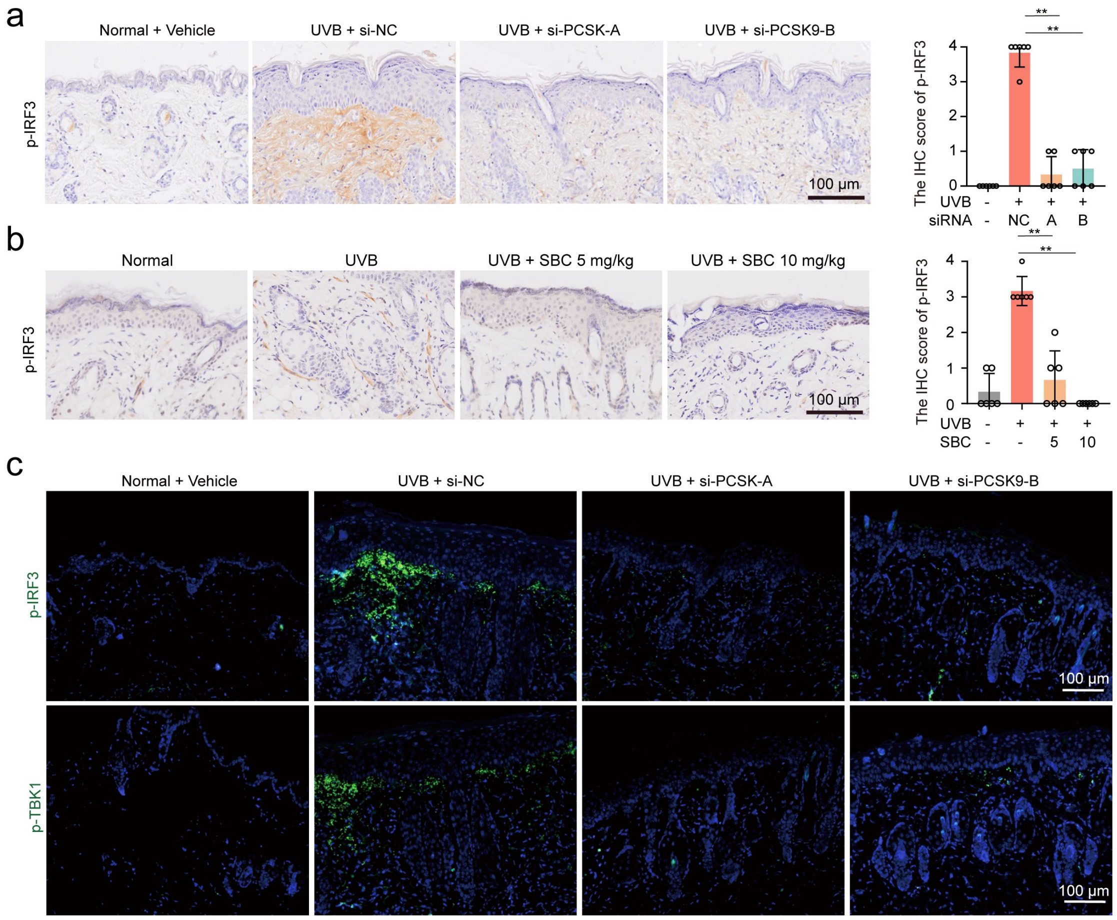

Supplementary Fig. s1 Topical application of PCSK9 siRNA and pharmacologic inhibition of PCSK9 reduced UVB-induced TBK1-IRF3 activation. (a) IHC staining of p-IRF3 expression on day 6. (b) IHC staining of p-IRF3 expression on day 6. (c) IF staining of p-IRF3 and p-TBK1.Data were shown as the means ± SD of five fields of view per mouse in every group, n=6 mice per group. *$P < 0.05$, **$P < 0.01$ vs. as indicated.

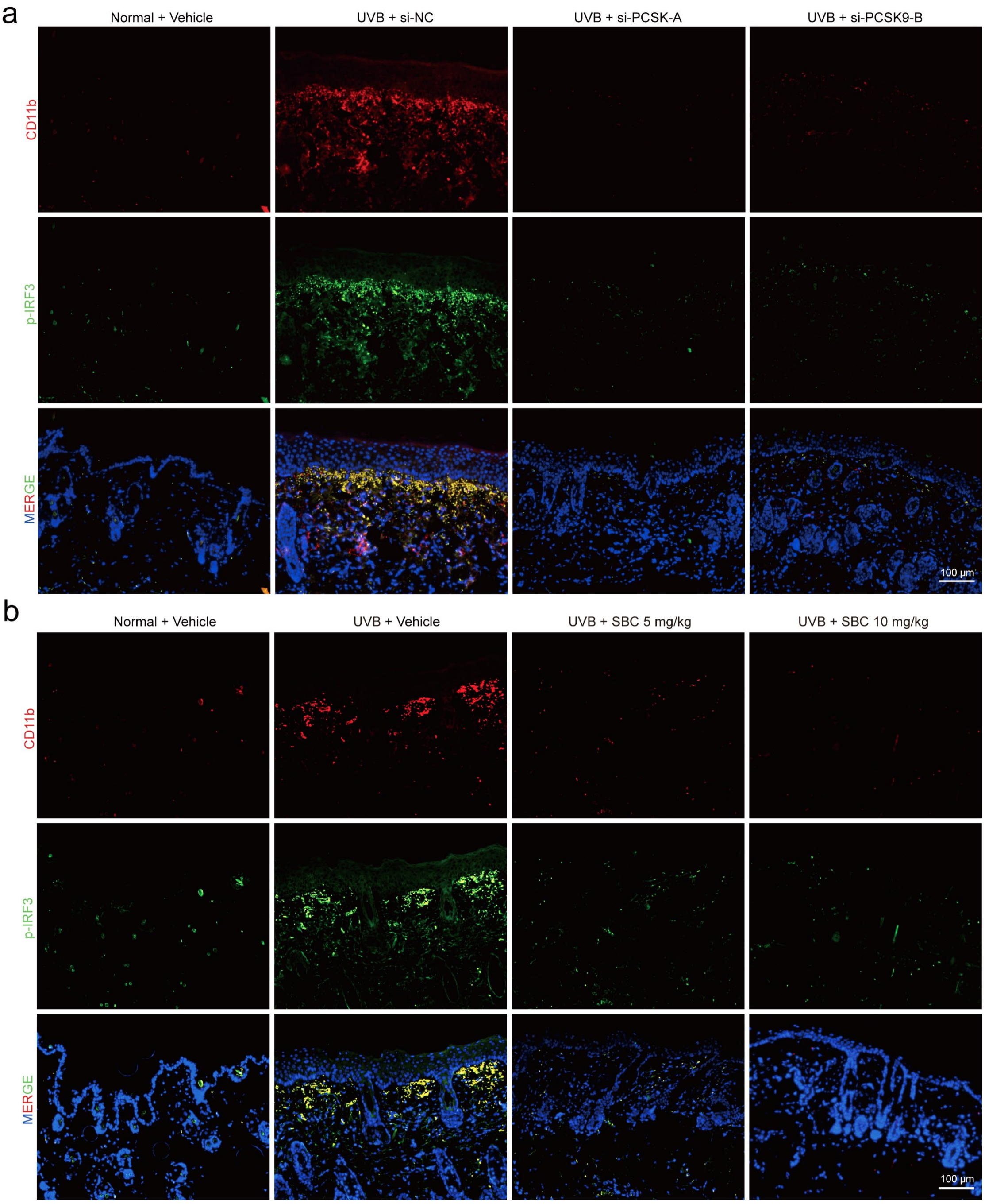

Supplementary Fig. s2 Topical application of PCSK9 siRNA and pharmacologic inhibition of PCSK9 reduced UVB-induced IRF3 activation located in macrophage marked by CD11b. (a) Representative image of CD11b and p-IRF3 expression in the experiment of topical application of PCSK9 on day 6. (b) Representative image of CD11b and p-IRF3 expression in the experiment of pharmacologic inhibition of PCSK9 on day 6. Scale bar: 100 μm. *$P < 0.05$, **$P < 0.01$ vs. as indicated.

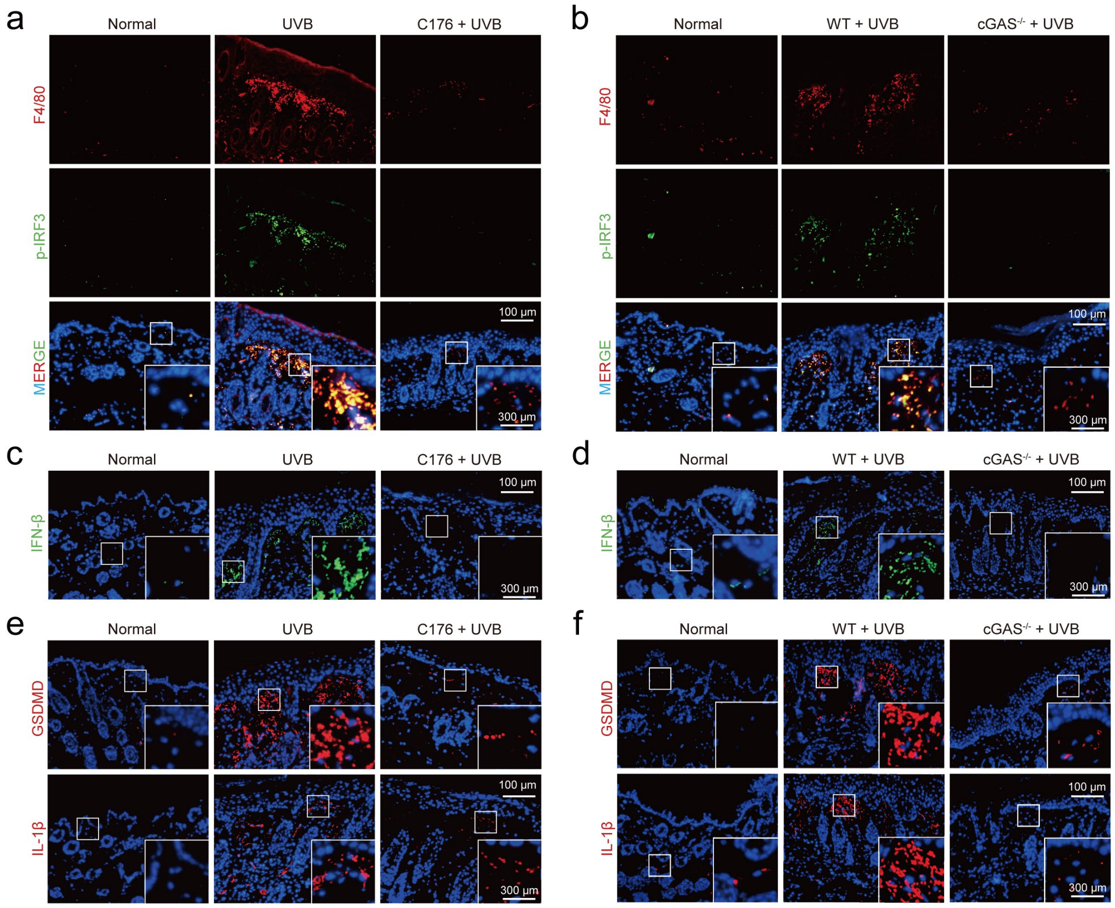

Supplementary Fig. s3 The deficiency of cGAS and pharmacologic inhibition of STING reduced UVB-induced damage. (a) Representative image of F4/80 and p-IRF3 expression in the experiment of pharmacologic inhibition of STING. (b) Representative image of F4/80 and p-IRF3 expression in the experiment of the deficiency of cGAS. (c) Representative image of IFN-β expression in the experiment of pharmacologic inhibition of STING. (d) Representative image of IFN-β expression in the experiment of the deficiency of cGAS. (e) Representative image of GSDMD and IL-1β expression in the experiment of pharmacologic inhibition of STING. (C) Representative image of GSDMD and IL-1β expression in the experiment of pharmacologic inhibition of STING.

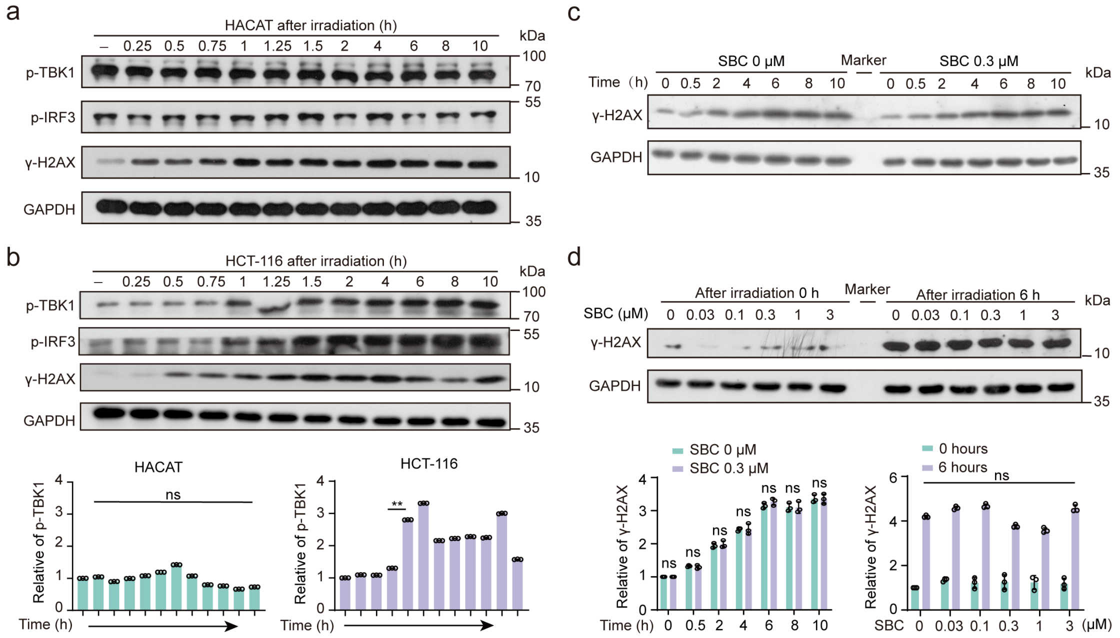

Supplementary Fig. s4 PCSK9 did not affect the activation of STING and DNA damage in HaCaT. (a) HaCaT cells were treated with UVB (50 mJ/cm^2), and cells were harvested at different hours after UVB irradiation. And the expression of the indicated proteins was measured using Western blot analysis. (b) HCT-116 cells were treated with UVB (50 mJ/cm^2), and cells were harvested at different hours after UVB irradiation. And the expression of the indicated proteins was measured using Western blot analysis. (c) and (d) HaCaT cells were treated with SBC (0.03, 0.1, 0.3, 1, 3 μM) or DMSO for 0 h, 0.5 h, 2 h, 4 h, 6 h, 8 h, 10 h, and the expression of the indicated proteins were measured using Western blot analysis. Data were shown as the means ± SD, n=3 per group. *P < 0.05, **P < 0.01 vs. as indicated. ns: not significant.

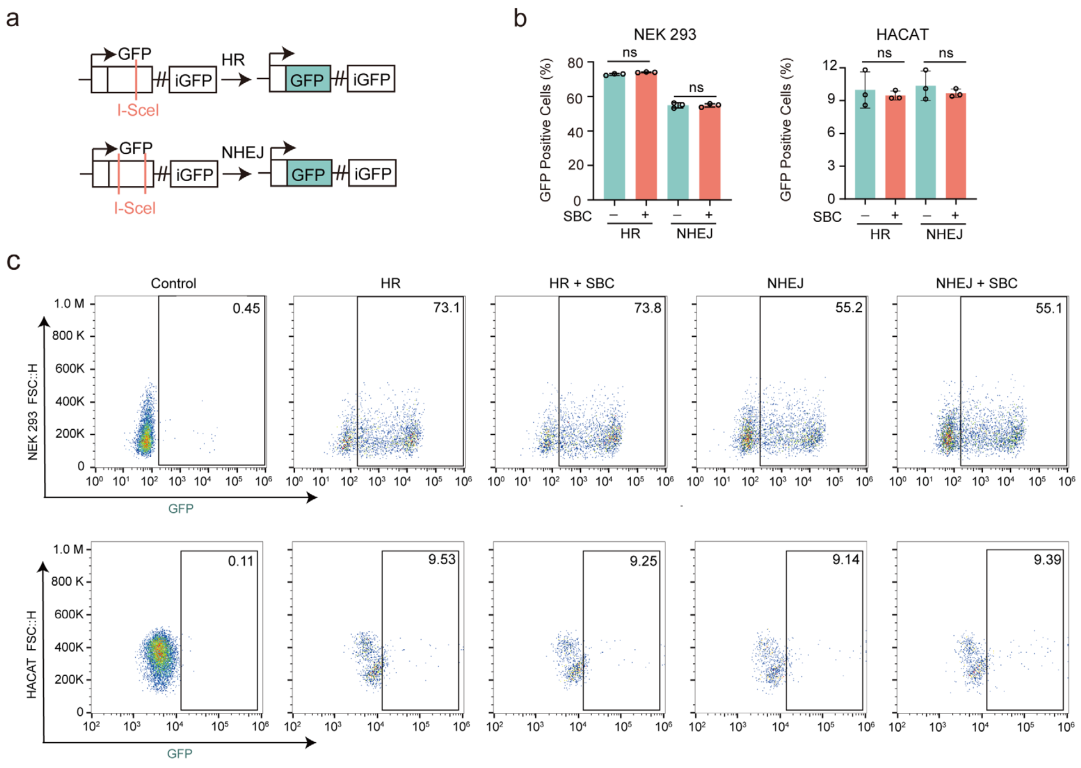

Supplementary Fig. s5 PCSK9 did not affect DNA damage repair in HaCaT. (a) Schematic of cell-based repair assay. (b) and (c) Obtained results showing the inhibition of PCSK9 did not have effectivity on the HR and NHEJ pathways. Representative FACS analysis of GFP-positive cells. Data were shown as the means ± SD, n=3 per group. **P* < 0.05, ***P* < 0.01 vs. as indicated. ns: not significant.

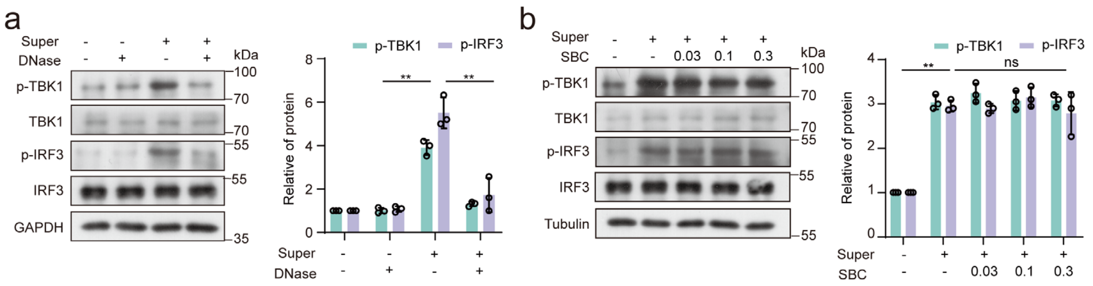

Supplementary Fig. S6 PCSK9 does not directly affect STING activation in macrophages. (a) Supernatant from HACAT treated with DNase failed to activate the STING pathway in PMA-differentiated THP-1 cells PMA-differentiated THP-1 cells. Supernatant from the HaCaT cells and DNase (2U/ml) were used to co-culture with PMA-differentiated THP-1 cells for 12 hours. (b) SBC110736 incubation with PMA-differentiated THP-1 cells didn't affect irradiated-HaCaT supernatant-triggered STING activation in PMA-differentiated THP-1 cells. Supernatant from the HaCaT culture and SBC110736 were used to co-culture with PMA-differentiated THP-1 cells for 12 hours. Data were shown as the means ± SD, n=3 per group. * $P < 0.05$, ** $P < 0.01$ vs. as indicated. Ns: not significant.
